# Galectin-9 Regulates Monosodium Urate Crystal-Induced Gouty Inflammation Through the Modulation of Treg/Th17 Ratio

**DOI:** 10.3389/fimmu.2021.762016

**Published:** 2021-10-28

**Authors:** Adel Abo Mansour, Federica Raucci, Anella Saviano, Samantha Tull, Francesco Maione, Asif Jilani Iqbal

**Affiliations:** ^1^ Institute of Cardiovascular Sciences (ICVS), College of Medical and Dental Sciences, University of Birmingham, Birmingham, United Kingdom; ^2^ Department of Clinical Laboratory Sciences, College of Applied Medical Sciences, King Khalid University, Abha, Saudi Arabia; ^3^ ImmunoPharmaLab, Department of Pharmacy, School of Medicine and Surgery, University of Naples Federico II, Naples, Italy

**Keywords:** galectin-9 (Gal-9), gout, MSU crystals, inflammation, cyto-chemokines

## Abstract

Gout is caused by depositing monosodium urate (MSU) crystals within the articular area. The infiltration of neutrophils and monocytes drives the initial inflammatory response followed by lymphocytes. Interestingly, emerging evidence supports the view that *in situ* imbalance of T helper 17 cells (Th17)/regulatory T cells (Treg) impacts the subsequent damage to target tissues. Galectin-9 (Gal-9) is a modulator of innate and adaptive immunity with both pro- and anti-inflammatory functions, dependent upon its expression and cellular location. However, the specific cellular and molecular mechanisms by which Gal-9 modulates the inflammatory response in the onset and progression of gouty arthritis has yet to be elucidated. In this study, we sought to comprehensively characterise the functional role of exogenous Gal-9 in an *in vivo* model of MSU crystal-induced gouty inflammation by monitoring *in situ* neutrophils, monocytes and Th17/Treg recruited phenotypes and related cyto-chemokines profile. Treatment with Gal-9 revealed a dose-dependent reduction in joint inflammation scores, knee joint oedema and expression of different pro-inflammatory cyto-chemokines. Furthermore, flow cytometry analysis highlighted a significant modulation of infiltrating inflammatory monocytes (CD11b^+^/CD115^+^/LY6-C^hi^) and Th17 (CD4^+^/IL-17^+^)/Treg (CD4^+^/CD25^+^/FOXP-3^+^) cells following Gal-9 treatment. Collectively the results presented in this study indicate that the administration of Gal-9 could provide a new therapeutic strategy for preventing tissue damage in gouty arthritic inflammation and, possibly, in other inflammatory-based diseases.

## Introduction

Inflammation is broadly defined as a host response to homeostatic imbalance triggered by conditions such as infection, tissue injury and noxious stimuli, including exposure to chemicals or radiation (1). One of the preliminary steps in the onset of inflammation is the recruitment of leukocytes to the site of injury or infection (2). Leukocyte recruitment is a highly intricate process comprising of several well-defined steps which include capture, rolling, adhesion/activation, intraluminal crawling and paracellular or transcellular transmigration (3). The infiltration of innate immune cells drives the initial inflammatory response followed by lymphocytes (3, 4). Emerging evidence supports the view that, in different inflammatory-based responses such as gouty arthritis, systemic imbalance of Th17/Treg induces their infiltration *in situ* and related damage to target tissues (5, 6). Tregs represent a small subset of T cells that control both innate and adaptive immune responses and are critical in maintaining self-tolerance and homeostasis (7). Treg have been shown to inhibit osteoclastogenesis by secreting immunosuppressive cytokines interleukin (IL)-10, transforming growth factor-β (TGF-β), and reducing gout bone damage (8, 9). Moreover, an increase in Treg levels has been shown to prevent an excessive immune response, while their loss is associated with major autoimmune diseases (10, 11). In contrast, Th17 cells are a subset of CD4^+^ T cells, characterised by IL-17 cytokine production that plays a powerful pro-inflammatory role in the immune system amplifying, rather than dampening, the progression of the inflammatory cascade (12). Indeed, the Th17/Treg balance provides a basis for understanding the immunological mechanisms that induce and regulate autoimmune and some inflammatory-based diseases (13).

Galectins are a family of carbohydrate-binding proteins that have a range of physiological functions, including regulation of cellular migration, cell cycle, proliferation, apoptosis and signal transduction (14). Galectins are found in the cytoplasm as well as the nucleus and are structurally characterised by the presence of one or two conserved ~130 amino-acid long carbohydrate recognition domains (CRDs) (15). To date, 15 genes encoding galectins have been identified in mammals, among which 12 have been identified in humans. The galectins can be broadly categorised into three sub-types: (a) prototype single CRD-galectins with the ability to form non-covalent homodimers (Gal-1, -2, -7, -10, -13, -14) in solution *via* non-covalent interaction, (b) chimeric-type comprising of a single CRD at the c-terminal and an n-terminal domain with an intrinsically disordered sequence (Gly-Pro-Tyr rich) which aids in oligomerisation (Gal-3) (16) and (c) tandem repeat-type which contain two unique CRD motifs at their n- and c-termini that are connected by a flexible linker of variable length (Gal-4, -8, -9, -12).

Here we focus on Gal-9 which was first identified as a novel eosinophil chemoattractant secreted by T cells (17). Among its major roles, in the context of inflammation, Gal-9 has been shown to have a range of pro- and anti-inflammatory functions dependent upon its expression and cellular localisation (18). It was shown that Gal-9 modulates the adaptive immune response by stimulating the maturation of antigen-presenting cells (APCs), specifically dendritic cells (19). This interaction elicits a selective production of IL-12 by dendritic cells which promotes the secretion of Th1 cytokines by CD4^+^ T cells (19). A study from Hafler et al., showed that this inflammatory activity observed in dendritic cells was dependent upon interaction with the T cell immunoglobulin mucin-3 (TIM-3) receptor (20). It has been further suggested that Gal-9 may have a beneficial role in the treatment of several inflammatory and autoimmune diseases (21, 22). Therapeutic application with recombinant Gal-9 was shown to inhibit the development of pathogenic Th17 cells and promote the expansion of Treg in a preclinical model of autoimmune arthritis (23).

In the context of gouty inflammation, MSU crystals promote the expansion of Th17 cells and their cognate cytokines. This inflammatory response can be inhibited by targeting IL-17 with neutralising antibodies, thereby reducing leukocyte infiltration into the inflamed tissue (5). As Gal-9 has previously been shown to both suppress the generation of Th17 (23, 24) and promote the induction of anti-inflammatory Treg cells (5, 23), we sought to investigate the action of exogenous Gal-9 in MSU-gouty inflammation in this current study. To address this, we assessed changes in both the innate and adaptive compartments (i.e. the numbers and types of cells infiltrating the joint and the severity of the disease) and local mediator production at the site of inflammation in a mouse model of gouty arthritis.

## Materials and Methods

### Reagents

Collagenase (Type VIII), dimethyl sulfoxide (DMSO), E-Toxate™ reagent from Limulus Polyphemus, fetal bovine serum (FBS), hyaluronidase, monosodium urate crystals (MSU), and RPMI-1640 cell medium were purchased from Sigma‐Aldrich Co. (Milan, Italy). Flow cytometry fixation and permeabilization buffer kit I, proteome profiler mouse cytokine array kit and recombinant mouse Gal-9 were purchased from R&D System (Milan, Italy). FACS buffer and conjugated antibodies from BioLegend (London, UK). Ficoll-Paque Plus (endotoxin tested, ≤ 0.12 EU/ml) was obtained from GE Healthcare Bio-Sciences AB (Uppsala, Sweden). Unless otherwise stated, all the other reagents were from BioCell (Milan, Italy).

### Animals

Mice care and experimental procedures were performed in accordance with international and national laws and policies. Mice experiments were designed in accordance with ARRIVE guidelines and the recommendations of the European Directive 2010/63/EU for animal experiments and the Basel Declaration, including the 3R concept (25, 26). Male mice CD-1 (age 10-14 weeks and weight 25-30 g) were obtained from Charles River (Milan, Italy) and preserved in a temperature- and humidity-controlled animal care facility, with a 12-h light/dark cycle, with *ad libitum* access to water and standard laboratory diet. All procedures were performed to reduce the number of animals used (n = 6 per group).

### Preparation of MSU Crystals

MSU crystals were prepared as previously described (27). Briefly, 800mg of MSU was dissolved in 155ml of boiling milli-Q water containing 5ml of NaOH, and the pH was adjusted to 7.2. The solution was cooled gradually by stirring at RT and crystals collected after centrifugation at 3000rpm for 5 mins at 4°C. The crystals were washed twice with 100% ethanol, dried, autoclaved (180°C for 2h), and weighed under sterile conditions. Crystals were resuspended in PBS by sonication and stored in a sterile environment until use. MSU crystals were confirmed as endotoxin-free by a commercial test kit of limulus polyphemus lysate assay (<0.01 EU/10 mg).

### MSU-Induced Acute Gouty Arthritis Model and Drug Administration

Acute gouty arthritis was induced by intra-articular (i.a.) administration of MSU crystals (200μg/20μl) into the right tibio-tarsal joint (ankle) of mice under isoflurane anaesthesia (28). Control (CTRL) group mice received an i.a. injection of sterile PBS (20μl). The successful establishment of the gouty arthritis model was judged by obvious swelling 2-3h after MSU injection (29). Animals from the groups treated with MSU received 1, 3 and 9µg (i.a.; in 20µl) of Gal-9 (single dose), 30 mins after MSU crystal administration. At the peak of inflammation (18h), tissues (knee joints) were collected, processed, and stored (-80°C) for further *ex vivo* analysis.

### Evaluation of Joint Scoring and Oedema

A first set of experiments were carried out to validate the dose-responsive effect of Gal-9. Mouse Joints were evaluated macroscopically using a scale ranging from 0 to 3, where 0 = no inflammation, 1 = mild inflammation, 2 = moderate inflammation and 3 = severe inflammation, in 0.25 increments. A score of 0.25 was given when the first signs of swelling and redness were present. Simultaneously, knee joint oedema was measured with a calliper before and after i.a. injection of MSU crystals or MSU crystals plus Gal-9 at the indicated times (4, 18, 24 and 48h). Knee joint oedema was determined by subtracting (for each mouse) the basal paw value from the value measured at each time point and expressed as Δ mm.

### Isolation and Characterisation of Joint-Infiltrating Cells

At the experimental end-point (18h) mice ankle joints were digested with hyaluronidase (2.4mg/ml) and collagenase (1mg/ml) in RPMI 1640 plus 10% FBS for 1h at 37°C, as previously described (30). Cells collected after digestion were filtered through a 70-μm nylon mesh filter (Becton Dickinson, Franklin Lakes, NJ, USA) and washed with RPMI 1640 plus 10% FBS. Subsequently, cells were washed in PBS for total cell counting prior to flow cytometry analysis.

### Cytokines and Chemokines Protein Array

Ankle joints were homogenised in ice-cooled Tris-HCl buffer (20mM, pH 7.4) containing 1mM EDTA, 1mM EGTA, 1mM PMSF, 1mM sodium orthovanadate, and one protease inhibitor tablet per 50ml of buffer. Protein concentration was determined by the BioRad protein assay kit (BioRad, Italy). According to the manufacturer’s instructions, equal volumes (1.5ml) of the pooled knee joint homogenates for all experimental conditions were then incubated with the pre-coated proteome profiler array membranes. Proteins were detected using the enhanced chemiluminescence detection kit and GE Healthcare Image Quant 400 software (GE Healthcare, Italy) and subsequently quantified using the GS 800 imaging densitometer software (Bio-Rad, Italy).

### *In Vitro* Treg Differentiation

Blood was collected from healthy donors with written and verbal informed consent and approval from the University of Birmingham Local Ethical Review Committee (ERN_18-0382). Human peripheral blood mononuclear cells (PBMCs) were isolated as previously described (31) and naive CD4^+^ T cells were isolated by negative selection using a commercial kit (Miltenyi, Biotec, Germany). Briefly, PBMCs were incubated with a biotin antibody cocktail for 5 mins at 4°C to bind with unwanted non-T cells, followed by incubation with anti-CD4^+^ microbeads for 10 mins at 4°C. Cells were added to MACS LS separation columns, and flow through containing the enriched CD4^+^ T cell fraction was collected.

On day one, CD4^+^ T cells were resuspended in 36ml of RPMI containing penicillin (50U/ml), streptomycin (50µg/m), L-glutamine (2mM) (Sigma), 5% FBS, and split into 6-well plates (6ml/well) and rested overnight in 5% CO_2_ at 37°C. 48-well plate was coated with anti-CD3 antibodies (1μg/ml, clone OKT3, BioLegend, UK) and incubated overnight at 4°C). On day two, CD4^+^ T cells were transferred into a 50 ml falcon tube and centrifuged at 250g for 10 mins at RT. The supernatant was discarded, and cell pellet was resuspended at a concentration of 8x10^5^ cells/ml in ImmunoCult™-XF T cell expansion medium (StemCell Technologies, Oxford, UK). Anti-CD3 antibody coated plates were washed with 150μl PBS without Ca^2+^ and Mg^2+^.

Separate plates were prepared by firstly adding either 50μl of blank T cell expansion medium or 50μl of a 4x concentration of a T cell polarization cocktail (final concentration: anti-CD28 1µg/ml (clone CD28.2, BioLegend), TGF-β 1ng/ml (R&D), IL-2 20U/ml (Roche). 50μl of blank T cell expansion medium or 50μl of Gal-9 was then added, followed by the addition of T cells (8x10^4^ cells/well). Cell suspensions were then mixed, transferred to the CD3-coated plate and incubated for 5 days at 37°C in 5% CO_2_. Treg expansion and purity was quantified by flow cytometry. Briefly, cells were washed in FACS buffer (PBS containing 1% BSA and 0.02% NaN_2_) and directly stained with the following conjugated antibodies (all from BioLegend, London, UK): CD3 (1:100, clone UCHT-1), CD4, (1:100, clone SK3), CD25 (1:50; clone AF-700) for 20 mins at 4°C. After washing, cells were fixated, permeabilized, and stained intracellularly with FOXP-3 antibody (1:50: clone 206D). At least 1×10^4^ cells were analysed per sample, and determination of positive and negative populations was performed based on the staining obtained with related IgG isotypes. After staining, samples were resuspended in PBS without Ca^2+^ and Mg^2+^ and analysed using the CyAn flow cytometer (Beckmann Coulter, USA). The flow cytometry data was analysed using Flowjo.

### Gal-9 Binding Human CD4^+^ T Cells

CD4^+^ T cells were incubated with or without recombinant Gal-9 (10nM) for 20 mins at RT. Cells were washed twice and incubated with anti-human Gal-9 antibody goat IgG (1:200, R&D Systems) and stained with the following conjugated antibodies (BioLegend, London, UK): CD4 (1:50, clone RPAT4), donkey anti-goat IgG (1:200, polyclonal) for 20 mins at 4°C. After washing, cells were fixated fixed in 1% PFA for storage before analysis with CyAn flow cytometer (Beckmann Coulter, USA).

### Human IL-10 ELISA

IL-10 Duo Set (R&D Systems, Abingdon, UK) was used to measure IL-10 levels in cell culture supernatants following manufacturer guidelines. Briefly, 96 well plates were coated with a human IL-10 capture antibody and incubated at RT overnight. Plates were washed 3 times with washing buffer to remove unbound antibodies and blocked for 1h at RT. 100µl of samples or standards (diluted with assay diluent) were added to the plate and incubated for 2h at RT. Plates were washed three times before the addition of 100µl of human IL-10 detection antibody for 1h at RT. Plates were washed three times before the addition of streptavidin-HRP for 20 mins at RT. Plates were washed three times before the addition of 100µl of substrate and left for 20 mins protected from light. Lastly, 50µl of stop (2M H_2_SO_4_) solution was added. A microplate reader was used to measure absorbance at 450nm.

### Flow Cytometry

Cells from digested joints were washed in FACS buffer (PBS containing 1% BSA and 0.02% NaN_2_) and directly stained with the following conjugated antibodies (all from BioLegend, London, UK): CD3 (1:200, clone 17A2), CD4 (1:200; clone GK1.5), CD25 (1:200; clone 3C7) for 60 mins at 4°C. After washing, cells were fixated, permeabilized, and stained intracellularly with IL-17A (1:200; clone TC11-18H10.1) and FOXP-3 antibody (1:200: clone MF-14). Moreover, for the characterization of joint-infiltration, cells were stained for CD45 (1:100; clone 30-F11), Ly6-C (1:100; clone HK1.4), Ly6-G (1:100; clone 1A8), CD11b (1:100; clone M1/70), CD115 (1:100; clone AFS98), prior to analysis. Th17, Treg, neutrophils, and patrolling/inflammatory monocytes were defined according to the flow cytometry procedure previously described (32). At least 1×10^4^ cells were analysed per sample, and determination of positive and negative populations was performed based on the staining obtained with related IgG isotypes (data not shown). Flow cytometry was performed on BriCyte E6 flow cytometer (Mindray Bio-Medical Electronics, Nanshan, China) using MRFlow and FlowJo software operation (28). Absolute numbers of positive cells for neutrophils (CD45^+^/Ly6-G^hi^/Ly6-C^hi^), monocytes (inflammatory: CD11b^+^/CD115^+^/Ly6-C^hi^; patrolling: CD11b^+^/CD115^+^/Ly6-C^lo^), Th17 (CD4^+^/IL-17^+^) and Treg (gated for CD4^+^ and then for CD25^+^/FOXP3^+^) were calculated converting the % of positive cells (for the mentioned staining) on the total number of leukocytes and CD4^+^ positive cells.

### Data and Statistical Analysis

All data are presented as means ± SEM and were analysed using students T-test or a one- or two-way ANOVA followed by Bonferroni’s or Tukey’s test for multiple comparisons. GraphPad Prism 8.0 software (San Diego, CA, USA) was used for analysis. Differences between means were considered statistically significant when P ≤ 0.05 was achieved. Sample size was chosen to ensure alpha 0.05 and power 0.8. Animal weight was used for randomisation and group allocation to reduce unwanted sources of variations by data normalisation. No animals and related *ex vivo* samples were excluded from the analysis. *In vivo* study was carried out to generate groups of equal size (n = 6 of independent values), using randomisation and blind analysis.

## Results

### Gal-9 Attenuates the Severity of MSU-Induced Gouty Inflammation

**Figure 1:**
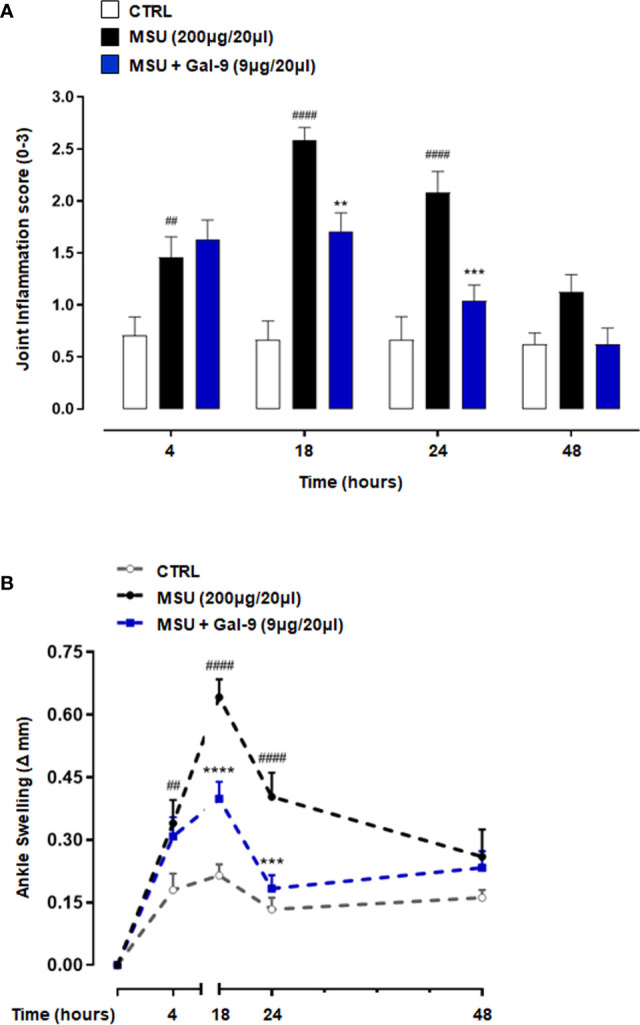
Gal-9 attenuates MSU crystals-induced gouty inflammation in mouse knee joints. Mice were treated with intra-articular (i.a.) dose of Gal-9 (9μg/20μl) or vehicle (PBS, 20μl) 30 mins after i.a. stimulation with MSU crystals (200μg/20μl) in the right knee joints. **(A)** Joint inflammation score (0-3 in increments of 0.25) and **(B)** joint inflammation oedema was evaluated at 4, 18, 24 and 48h after the stimulus with MSU. Data (expressed as joint inflammation score and Δ increase of knee joints mm respectively) are presented as means ± SEM of n = 6 mice per group. Statistical analysis was conducted by one- or two-way ANOVA followed by Bonferroni’s for multiple comparisons. ^##^P ≤ 0.01, ^####^P ≤ 0.0001 *vs* Ctrl group; **P ≤ 0.01, ***P ≤ 0.001, ****P ≤ 0.0001 *vs* MSU group.

To investigate the potential anti-inflammatory effect of Gal-9, we used a mouse model in which MSU crystals were injected into knee joints, to mimic the etiologic cause of human gouty inflammation (33). MSU crystals injection (200µg/20µl) evoked an intense and robust joint inflammatory score (peak at 18h) that was dose-dependently attenuated by Gal-9 (1-9µg/20µl) administration (**Supplementary Figure 1A**), with maximum inhibition observed at a dose of 9µg/20µl (**Figure 1A**). In addition to joint inflammation scores, we evaluated ankle swelling and found Gal-9 treatment (9µg/20µl) significantly reduced ankle swelling between 18 and 24 h (**Figure 1B**), indicating an enhanced resolution profile in the presence of Gal-9. A significant reduction was also observed at 18 h at a concentration of 3µg/20µl, however at a lower concentration of 1μg/20µl no appreciable effects were observed on joint scores or swelling (**Supplementary Figure 1**). Based on the results obtained, we selected the most effective dose of Gal-9 (9µg/20µl) for all subsequent experiments.

### Gal-9 Modulates, *In Situ*, the Recruitment of Leukocytes

**Figure 2:**
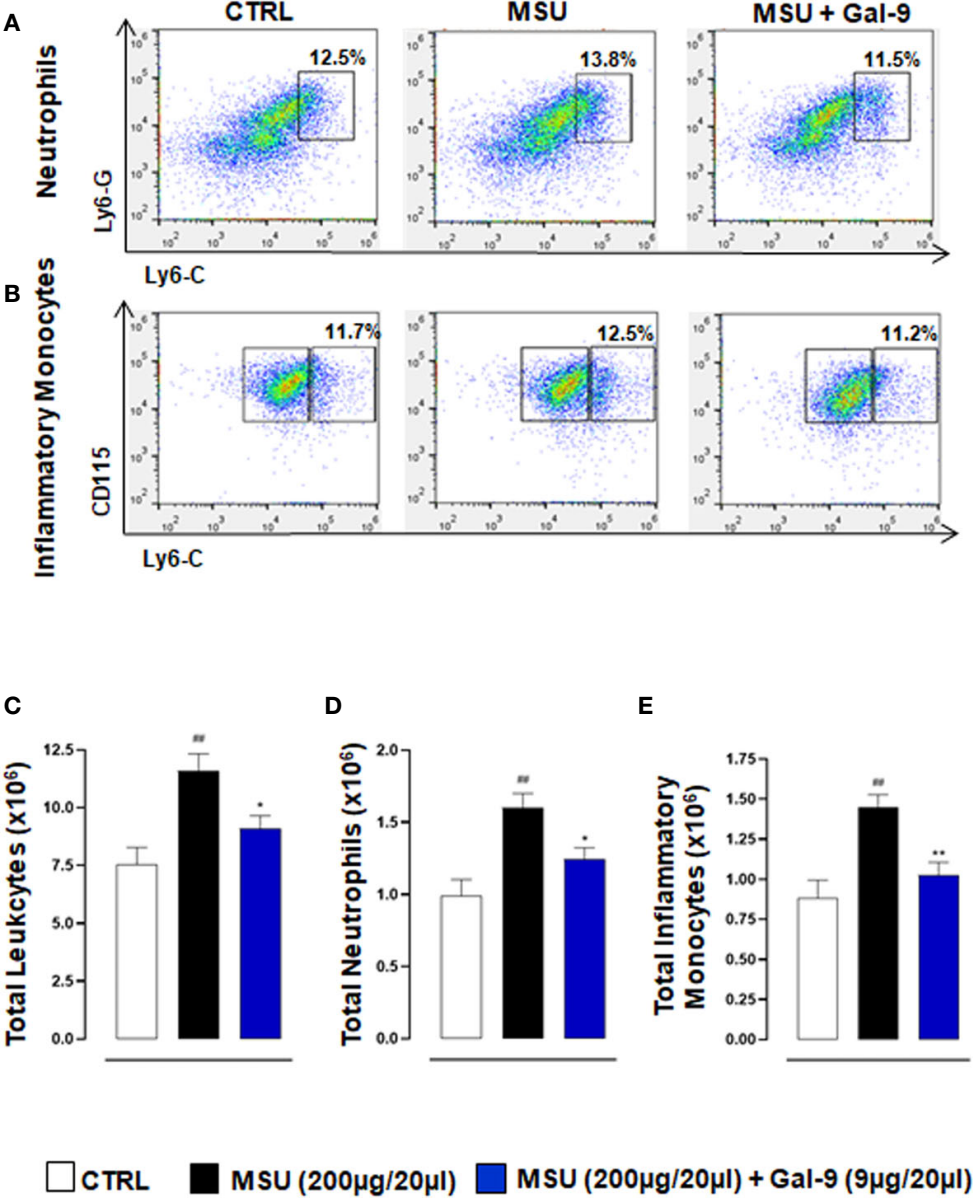
Gal-9 modulates the recruitment of innate immune cells. Flow cytometry analysis was employed to determine *in situ* neutrophil and monocyte subsets. At the peak of inflammatory reaction (18h), ankle joints were digested, and single cell suspensions were obtained. Flow cytometry strategy applied to identify neutrophils **(A)**, and monocytes **(B)**, after Gal-9 treatment are shown. Cells were washed and stained with: CD45, LY6-C, LY6-G, CD11b, and CD115/CD45^+^ cells were plotted for LY6-C and LY6-G expression to identify CD45^+^/LY6-C^hi^/LY6-G^hi^ as neutrophils **(A)**. CD11b^+^ cells were plotted for LY6-C and CD115 expression to distinguish CD11b^+^/CD115^+^/LY6-C^lo^ patrolling monocytes from CD11b^+^/CD115^+^/LY6-C^hi^ inflammatory monocytes **(B)**. **(C)** Total infiltrated leukocytes, **(D)** neutrophil and **(E)** inflammatory monocytes were quantified in the different experimental conditions. Representative FACS plots of three independent experiments with similar results are shown. Values are presented as means ± SEM of n = 6 mice per group. Statistical analysis was conducted by one-way ANOVA followed by Bonferroni’s for multiple comparisons. ^##^P ≤ 0.01 *vs* Ctrl group; *P ≤ 0.05, **P ≤ 0.01 *vs* MSU group.

During the onset and resolution phases of gouty arthritis, a major hallmark of disease pathogenesis is the infiltration of immune cells, with mainly neutrophils and inflammatory monocytes in the early phases (34, 35) followed by CD4^+^ T cells in the latter (9, 36). We, therefore, characterised the phenotype of recruited cells following MSU injection and Gal-9 administration. Flow cytometry was employed to determine neutrophil, monocytes, Th17 and Treg populations in single cell suspensions following digestion of knee joint tissues harvested from the 18h time-point. To identify leukocyte subpopulations, total cells followed by single-cells were gated, and CD45 (pan leukocyte/immune cell marker) in combination with CD11b (myeloid marker) and CD4 (accessory protein for MHC class-II antigen/T-cell receptor interaction) were used to identify neutrophils, monocytes and T cells. Neutrophils were further characterised as CD45^+^/Ly6-G^hi^/Ly6-C^hi^ (**Figure 2A**) and monocytes were delineated based upon Ly6-C and CD115 expression to distinguish CD11b^+^/CD115^+^/Ly6-C^lo^ patrolling monocytes from CD11b^+^/CD115^+^/Ly6-C^hi^ inflammatory monocytes (**Figure 2B**). In agreement with our previous studies (5), injection of MSU crystals resulted in a significant increase in the total number of leukocytes recruited to joints when compared to CTRL (**Figure 2C**). A significant reduction in total leukocytes was observed in mice administered with Gal-9 when compared to MSU alone (**Figures 2C**). Moreover, mice injected with MSU crystals alone compared to CTRL showed significant recruitment of neutrophils (**Figure 2D**) and inflammatory monocytes (**Figure 2E**). In line with total leukocyte counts, treatment with Gal-9 significantly reduced neutrophil and inflammatory monocytes levels when compared to MSU-injected mice alone, in terms of cell percentages as well as absolute numbers (**Figures 2A, B, D, E**). In all experimental conditions, no significant differences were found in the proportion of patrolling monocytes (data not shown).

**Figure 3:**
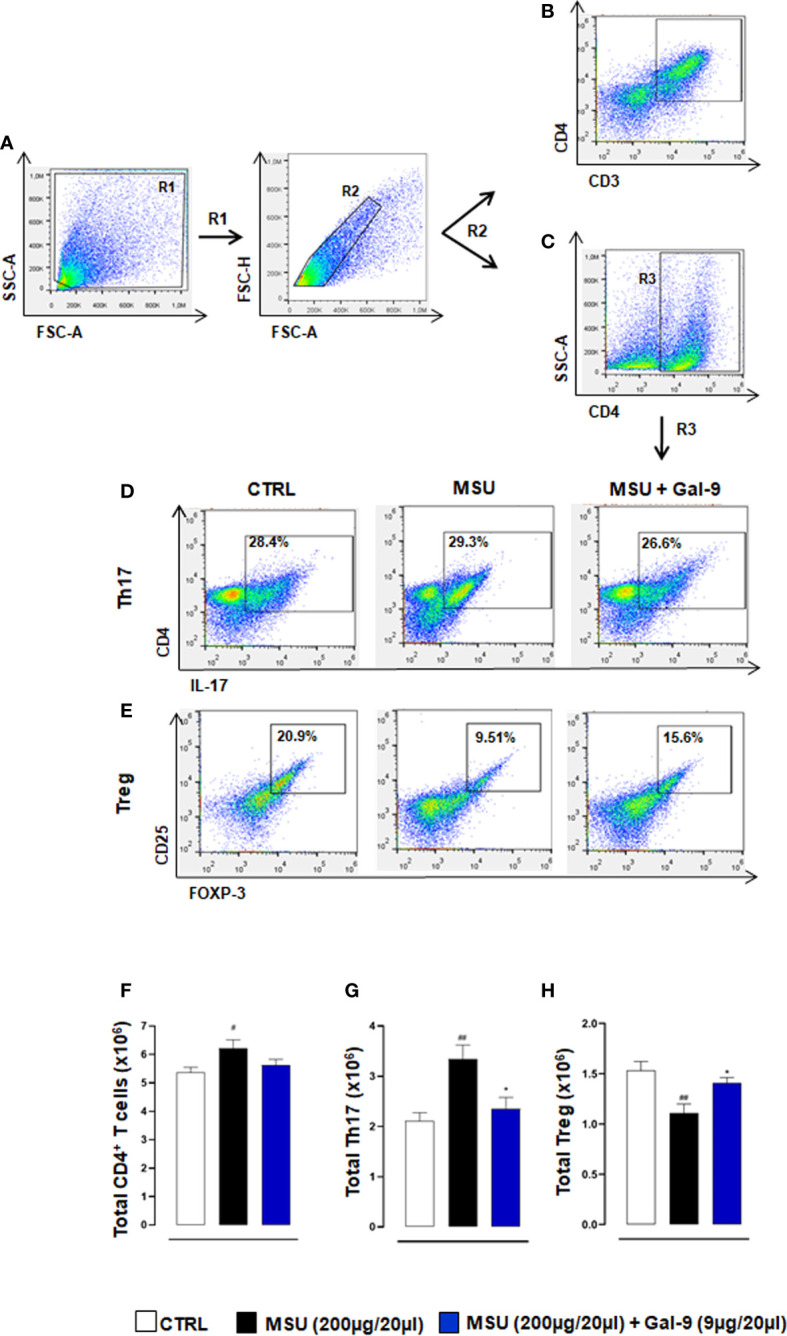
Gal-9 sustains local Treg levels. Flow cytometry analysis was employed to determine *in situ* levels of CD4^+^ T cells, Th17 and Tregs subsets. At the peak of the inflammatory reaction (18h), ankle joints were digested, and single cell suspensions were obtained. Flow cytometry strategy applied to identify the modulation of CD3/CD4 **(A, B)**, CD4^+^ T cells **(A, C)**, Th17 **(A, C, D)** and Tregs **(A, C, E)** after Gal-9 treatment are shown. Cells were washed and stained with: CD3, CD4, CD25, and intracellular antibodies IL-17A and FOXP-3. Th17 and Treg populations were defined as CD4^+^/IL-17^+^ **(A, C, D)** and CD4^+^/CD25^+^/FOXP-3^+^ **(A, C, E)** respectively. **(F)** Total CD4^+^ T cells, **(G)** Th17 and **(H)** Tregs were quantified in the different experimental conditions. Representative FACS plots of three independent experiments with similar results are shown. Values are presented as means ± SEM of n = 6 mice per group. Statistical analysis was conducted by one-way ANOVA followed by Bonferroni’s test for multiple comparisons. ^#^P ≤ 0.05, ^##^P ≤ 0.01 *vs* Ctrl group; *P ≤ 0.05 *vs* MSU group.

To clarify if potential differences in inflammatory/resolution profiles *in situ* were a result of alterations in T cell subset ratios, we stained knee joint homogenates for CD3/CD4 (**Figures 3A, B**) and CD4 to identify Th17 and Treg populations defined as CD4^+^/IL-17^+^ and CD4^+^/CD25^+^/FOXP-3^+^ respectively (**Figures 3A, C–E**). MSU-injected mice displayed a significant increase in total CD4^+^ T cells compared to control mice (**Figure 3F**). This increase was associated with elevated Th17 cells and reduced Treg levels when compared to CTRL (**Figures 3G, H**). In stark contrast mice treated with Gal-9 displayed a significant reduction in Th17 cells and sustained levels of Tregs (**Figures 3G, H**). We also analysed the circulating Th17/Tregs profile but did not see differences between groups after Gal-9 injection (data not shown).

### Gal-9 Treatment Reduces Local Production of Pro-Inflammatory Cyto-Chemokines

**Figure 4:**
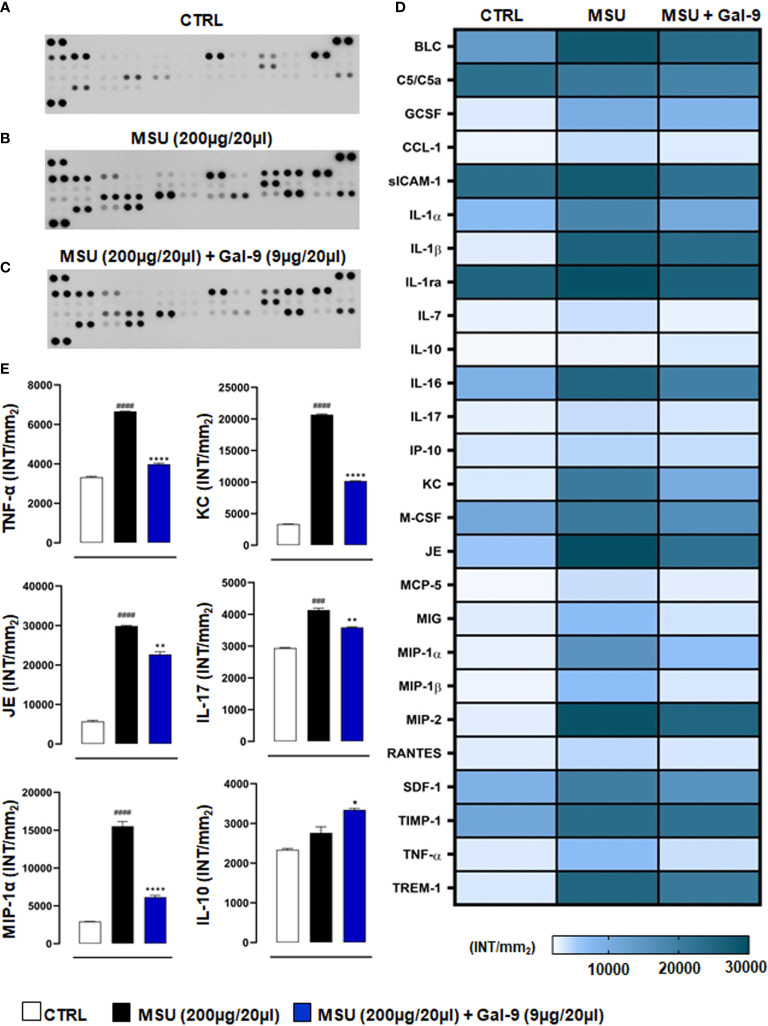
Gal-9 decreases the release of cyto-chemokines in knee joints. Inflammatory fluids obtained from knee joint homogenates at 18h time-point, were assayed using a proteome profiler cytokine array for **(A)** CTRL, **(B)** MSU and **(C)** MSU + Gal-9 group. Densitometric analysis is presented as heatmap **(D)**. Thereafter, IL-10, IL-17, KC, JE, MIP-1α and TNF-α were extrapolated from heatmap and represented graphically **(E)**. Data (expressed as INT/mm^2^) are presented as means ± SEM. of positive spots of three separate independent experiments run each with n = 6 mice per group pooled. Statistical analysis was performed by using two-way ANOVA followed by Bonferroni’s test for multiple comparisons. ^###^P ≤ 0.001, ^####^P ≤ 0.0001 *vs* Ctrl group; *P ≤ 0.05, **P ≤ 0.01, ****P ≤ 0.0001 *vs* MSU group.

Considering the importance of pro-inflammatory mediators in driving recruitment and local immune cell activation in disease onset and progression (37), we next sought to determine the effect of Gal-9 treatment on local cytokine and chemokine production. As shown in **Figure 4**, knee joint homogenates (collected at 18h time-point) obtained from MSU-administered mice showed a large increase of pro-inflammatory cyto-chemokines compared to CTRL group (**Figures 4A, B**). Interestingly, after Gal-9 treatment, there was a significant decrease in a range of mediators (**Figure 4C**). According to cellular profile previously characterised by FACS, densitometric analysis, presented as a heatmap (**Figure 4D**), revealed that the Gal-9 treated group had a significant (P ≤ 0.0001) modulation in the following factors: B lymphocyte chemoattractant (BLC), component 5a (c5/c5a), soluble intercellular adhesion molecule-1 (sICAM-1), IL-1α, IL-1β, IL-1ra, IL-16, keratinocyte chemoattractant (KC), macrophage colony-stimulating factor (M-CSF), monokine induced by interferon-γ (MIG), macrophage inflammatory protein (MIP)-1α, MIP-1β, MIP-2, IL-1β stromal cell-derived factor-1 (SDF-1), TNF-α and triggering receptor expressed on myeloid cells-1 (TREM-1). To a lesser extent, we also observed a modulation (P ≤ 0.01) of granulocyte colony-stimulating factor (G-CSF), IL-17 and junctional epithelium (JE) compared to MSU group. A minor inhibitory profile (P ≤ 0.05) was found for IL-7, IL-10, monocytes chemoattractant protein-5 (MCP-5), and metallopeptidase inhibitor-1 (TIMP-1) (**Figure 4D**). Major cytokines and chemokines involved in driving disease (IL-10, IL-17, KC, JE, MIP-1α and TNF-α) have been extrapolated from the heatmap and represented graphically (**Figure 4E**).

### Gal-9 Promotes the Differentiation of Naïve CD4 T Cell Towards Treg Phenotype

**Figure 5:**
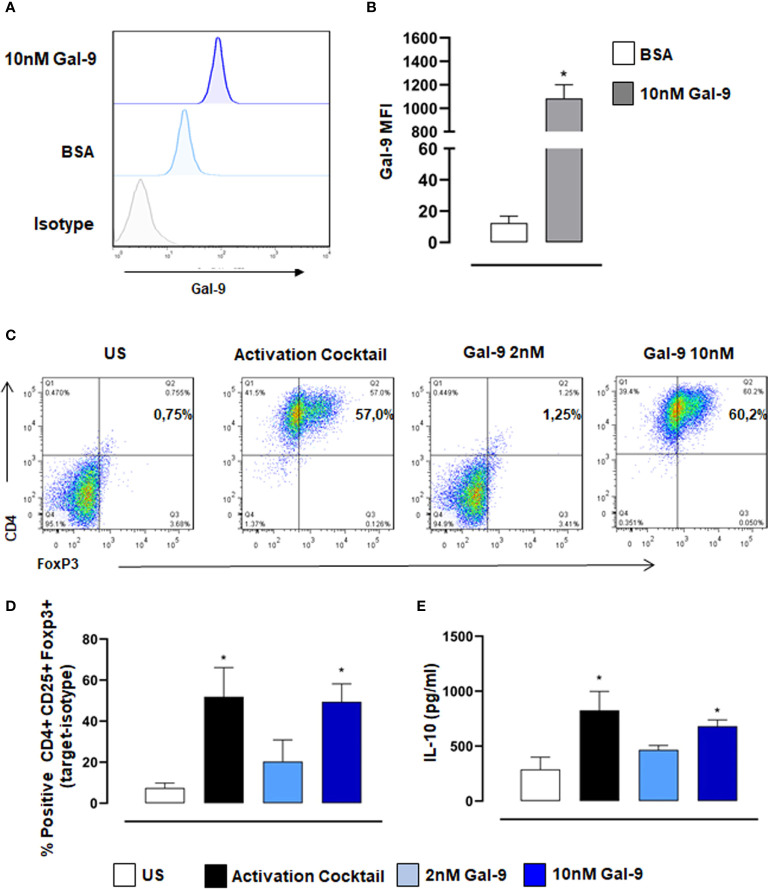
Gal-9 induces Treg differentiation of naïve human CD4^+^ T cells. CD4^+^ T cells were isolated from PBMCs and incubated with 10nM Gal-9 before staining with anti-Gal-9. **(A)** Representative histogram showing exogenous Gal-9 binding in CD4^+^ cells compared to isotype control levels. **(B)** Quantification of Gal-9 protein levels using median fluorescence intensity (MFI) of Gal-9. Data are expressed as mean ± SEM (n=4). Statistical analysis was performed with Students T-test; *P < 0.05 vs BSA. Naïve CD4^+^ T cells were differentiated with activation cocktail (CD3/CD28, IL2, TGFβ) or Gal-9 alone for 5 days. **(C, D)** Flow cytometry was used to measure % positive expression of CD4^+^,CD25^+^ and Foxp3^+^. **(E)** Cell culture supernatants were collected, and IL-10 levels were measured by ELISA. Data are expressed as mean ± SEM (n=3). Statistical analysis was performed with one-way ANOVA with Tukey multiple comparisons; *P < 0.05.

Previous reports and data in this study support the view that therapeutic application of Gal-9 *in vivo* promotes Treg induction which dampens inflammation and promotes resolution. We, therefore, carried out *in vitro* assays with purified human naive CD4^+^ T cells to test the capacity of Gal-9 to induce Treg differentiation. Initially, we confirmed with flow cytometry that exogenously added Gal-9 binds to CD4^+^ T cells (**Figures 5A, B**). Moreover, we found that Gal-9 alone did indeed promote Treg differentiation, defined as CD4^+^/FoxP3^+^, and that this effect was concentration-dependent, with 10nM far more efficient (similar degree to the activation cocktail) compared to 2nM Gal-9 (**Figures 5C, D**). We also measured IL-10 release into cell culture supernatants, a functional readout of Tregs, and found a similar significant increase in cells treated with 10nM Gal-9 compared to control (**Figure 5E**).

## Discussion

In the presence of sodium, uric acid from purine metabolism precipitates as MSU needles and can contribute to the development of gouty arthritis, characterised by redness, heat, and swollen joints (37). Analyses of synovial fluids and tissue sections of patients suffering from gout has revealed the presence of granuloma and neutrophil extracellular traps (NETs) formation which, after prolonged exposure, carries the risk for the development of chronic inflammation (38). The pathogenesis of chronic gouty arthritis is intricate, and its progression involves a variety of immunological factors. T-cell dysfunction (in particular the imbalance between Treg/Th17) plays an essential role in the occurrence and development of disease. Indeed, restoring the protective levels of Treg (36) or reducing the self-perpetuating inflammatory Th17 subset (5) represents one of the key immunological features in gouty arthritis.

Findings from several studies have shown that expression profile of Gal-9 is highly elevated in many inflammatory autoimmune diseases, such as systemic lupus erythematosus (39), rheumatoid arthritis (RA) (40, 41), and systemic sclerosis (42). Gene polymorphisms in LGALS2, LGALS3 and LGALS9 have been linked with predisposition to RA (43). Studies have also proposed the participation of Gal-9 in the immunopathogenesis of systemic inflammatory processes (44) and as a pro-active serum checkpoint molecule in RA (45).

The current study used an MSU-driven model of acute gouty arthritis and, in that context, our results showed that injection of MSU crystals (200µg/20µl) induced ankle swelling (with a peak at 18h) and inflammation. Therapeutic application of Gal-9 (9µg/20µl) significantly reduced ankle swelling observed at 18h and 24h after MSU crystal administration. Moreover, our results showed that Gal-9 administration reduced inflammatory cell infiltration and levels of pro-inflammatory mediators produced by neutrophils/inflammatory monocytes (e.g. IL-1α/β, TNF-α, JE, KC) and by Th17/Treg cell subtypes (e.g. IL-10, IL-17, G-CSF) at the local site of inflammation, which suggests an anti-inflammatory mode of action for Gal-9 in this model.

To investigate which types of immune cells were preferentially recruited in response to MSU crystals and those to MSU in combination with Gal-9 treatment, we employed flow cytometry. Cytometric data showed that neutrophils and inflammatory monocytes were the main hallmarks of the MSU group compared to CTRL, with a significant increase in Th17 infiltration. In contrast, Gal-9 treatment maintained the total CD4^+^ and Treg numbers at CTRL levels, whilst abolishing any MSU-induced increase in Th17 numbers, thereby maintaining a Th17/Treg balance. Furthermore, treatment with Gal-9 clearly suppressed the initial innate immune response, with a significant reduction observed in neutrophil and inflammatory monocyte recruitment. This coupled with the reduction in local inflammatory mediators could have a major impact on the secondary wave of T cell recruitment and subsequent amplification of the inflammatory response. Raucci and colleagues recently demonstrated the importance of T cells in driving pathogenesis in this model of gouty arthritis (5). They highlighted that suppressing Th17 cells using a neutralizing antibody against IL-17, resulted in elevated circulating Treg levels, which in turn accelerated inflammation resolution, thus indicating a potential modulatory role for Tregs in MSU gouty inflammation. In another experimental model of autoimmune induced arthritis, therapeutic application of Gal-9 was shown to inhibit the development of Th17 cells and promote the expansion of Tregs cells (23). The authors showed that Gal-9 suppressed arthritis in a dose-dependent fashion by inhibiting the expression of pro-inflammatory cytokines, mainly IL-17, IL-12, and IFN-γ in the joints. Previous studies have shown that Gal-9 selectively induces apoptosis in Th1 and Th17 cells through its interaction with TIM-3 (46, 47). This could also be a potential mode of action in this current study and warrants further investigation.

In human settings, Gal-9 expression and role in autoimmune arthritis has been previously demonstrated, with elevated expression of this protein in the synovial fluid of RA patients compared to those with osteoarthritis (21). More recently a study from Sun et al., demonstrated that Gal-9 expression in T cells positively correlated with disease activity and could be potentially used as a novel biomarker for evaluating RA activity and therapeutic effect (40). Gal-9 modulation of RA *via* the regulation of synovial fibroblast activity/viability has been shown to be complex, with endogenous Gal-9 protecting synovial fibroblasts against apoptosis (48), while exogenous Gal-9 has been shown to induce apoptosis in fibroblast-like synoviocytes in RA patients (21). Here, we selectively isolated human naive CD4^+^ T cells and treated them with Gal-9, to assess the ability of Gal-9 to drive human T cell differentiation. In line with previous studies (49, 50), we found that Gal-9 alone was effective at inducing Treg differentiation in a dose-dependent manner. Moreover, there was also a dose-dependent increase in IL-10 levels in supernatants from Gal-9 treated T cell cultures, suggesting a specific role for Gal-9 in inducing Treg functions (i.e., IL-10 production).

In conclusion, our findings suggest that Gal-9 has a crucial role in regulating the acute inflammation and resolution associated with gouty arthritis through sustaining anti-inflammatory Treg populations locally at the site of inflammation, while simultaneously reducing Th17 levels. Administration of Gal-9 could provide a new therapeutic strategy for preventing tissue damage associated with gouty arthritis.

## Data Availability Statement

The raw data supporting the conclusions of this article will be made available by the authors, without undue reservation.

## Ethics Statement

Blood was collected from healthy donors with written and verbal informed consent and approval from the University of Birmingham Local Ethical Review Committee (ERN_18-0382). The patients/participants provided their written informed consent to participate in this study. All animal care and experimental procedures were carried out in compliance with the international and national law and policies and approved by the Italian Ministry of Health. Animal studies were reported in compliance with the ARRIVE guidelines and with the recommendations made by EU Directive 2010/63/EU for animal experiments.

## Author Contributions

AM, FR, AS, ST, and FM performed experiments. AM, FR, FM, and AI analysed results and made the figures. AI and FM designed the research. AM, FR, FM, and AI wrote the paper. All authors contributed to the article and approved the submitted version.

## Funding

AM is supported by a KSA government-funded scholarship. FR is supported by a University of Naples Federico II PhD scholarship in Pharmaceutical Sciences. AS is supported by a Dompé Farmaceutici S.p.A fellowship for PhD programme in “Nutraceuticals, functional foods and human health” (University of Naples Federico II). AI is supported by a Birmingham Fellowship.

## Conflict of Interest

The authors declare that the research was conducted in the absence of any commercial or financial relationships that could be construed as a potential conflict of interest.

## Publisher’s Note

All claims expressed in this article are solely those of the authors and do not necessarily represent those of their affiliated organizations, or those of the publisher, the editors and the reviewers. Any product that may be evaluated in this article, or claim that may be made by its manufacturer, is not guaranteed or endorsed by the publisher.

## Supplementary Material

Supplementary Figure 1 Mice were treated with Gal-9 (1-9μg/20μl) or vehicle (PBS, 20μl) 30 mins after i.a. stimulation with MSU crystals (200μg/20μl) in the right knee joints. **(A)** Joint inflammation score (0-3 in increments of 0.25) and **(B)** joint inflammation oedema was evaluated at 4, 18, 24 and 48h after MSU. Data (expressed as joint inflammation score and Δ increase of knee joints mm respectively) are presented as means ± SEM of n = 6 mice per group. Statistical analysis was conducted by one- or two-way ANOVA followed by Bonferroni’s for multiple comparisons. ^#^P ≤ 0.05, ^##^P ≤ 0.01, ^####^P ≤ 0.0001 *vs* Ctrl group; *P ≤ 0.05, **P ≤ 0.01, ***P ≤ 0.001, ****P ≤ 0.0001 *vs* MSU group.

## Abbreviations

APCs, antigen-presenting cells; BLC, B lymphocyte chemoattractant; c5/c5a, component 5a; CRDs, carbohydrate recognition domains; CTRL, Control; DMSO, dimethyl sulfoxide; G-CSF, granulocyte colony-stimulating factor; Gal-, Galectin-; FBS, fetal bovine serum; i.a., intra-articular; IL-, interleukin-; JE, junctional epithelium; KC, keratinocyte chemoattractant; MCP-5, monocytes chemoattractant protein-5; M-CSF, macrophage colony-stimulating factor; MAPK, mitogen-activated protein kinase; MIG, monokine induced by interferon-γ MIP, macrophage inflammatory protein; MSU, monosodium urate; NETs, neutrophil extracellular traps; PDIs, protein disulfide isomerase; RA, rheumatoid arthritis; SDF-1, IL-1β stromal cell-derived factor-1; sICAM-1, intercellular adhesion molecular-1; TGF-β, transforming growth factor-β; Th17, T helper 17 cells; TIM-3, T cell immunoglobulin mucin-3; TIMP-1, metallopeptidase inhibitor-1; TNF-α, tumor necrosis factor-α; Treg, regulatory T-cells; TREM-1, triggering receptor expressed on myeloid cells-1.
