# Supplementary figures and images for: Galectin-9 Regulates Monosodium Urate Crystal-Induced Gouty Inflammation Through the Modulation of Treg/Th17 Ratio
Source: Front Immunol. 2021 Oct 28;12:762016. doi: 10.3389/fimmu.2021.762016 (PMC8581207; doi:10.3389/fimmu.2021.762016)

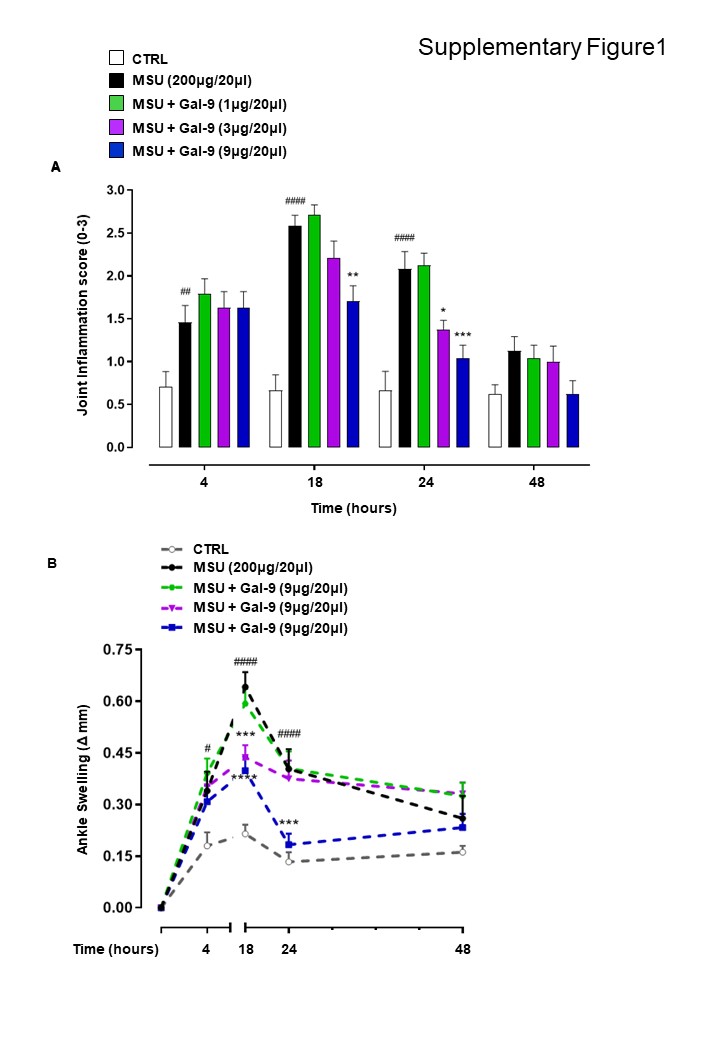

Supplement: Supplementary Figure 1 — Mice were treated with Gal-9 (1-9μg/20μl) or vehicle (PBS, 20μl) 30 mins after i.a. stimulation with MSU crystals (200μg/20μl) in the right knee joints. (A) Joint inflammation score (0-3 in increments of 0.25) and (B) joint inflammation oedema was evaluated at 4, 18, 24 and 48h after MSU. Data (expressed as joint inflammation score and Δ increase of knee joints mm respectively) are presented as means ± SEM of n = 6 mice per group. Statistical analysis was conducted by one- or two-way ANOVA followed by Bonferroni’s for multiple comparisons. #P ≤ 0.05, ##P ≤ 0.01, ####P ≤ 0.0001 vs Ctrl group; *P ≤ 0.05, **P ≤ 0.01, ***P ≤ 0.001, ****P ≤ 0.0001 vs MSU group. [file Image_1.jpeg]
